# Recovery of gastrointestinal functional after surgery for abdominal tumors: A narrative review

**DOI:** 10.1097/MD.0000000000040418

**Published:** 2024-11-01

**Authors:** Gui-Sheng Xie, Liang Ma, Jian-Hong Zhong

**Affiliations:** a General Surgery Department, The Third Affiliated Hospital of Guangxi Medical University, Nanning, China; b Hepatobiliary Surgery Department, Guangxi Medical University Cancer Hospital, Nanning, China; c Key Laboratory of Early Prevention and Treatment for Regional High Frequency Tumor (Guangxi Medical University), Ministry of Education, Nanning, China; d Guangxi Key Laboratory of Early Prevention and Treatment for Regional High Frequency Tumor, Nanning, China.

**Keywords:** complications, length of hospital stay, postoperative ileus

## Abstract

Postoperative gastrointestinal dysfunction, including temporary nonmechanical suppression of gastrointestinal motility (known as postoperative ileus), occurs in about 10% surgeries of abdominal tumors. Since these complications can prolong hospitalization and affect eating, it is important to understand their risk factors and identify effective interventions to manage or prevent them. The present review comprehensively examined the relevant literature to describe risk factors for postoperative ileus and effective interventions. Risk factors include old age, open surgery, difficulty of surgery, surgery lasting longer than 3 hours, preoperative bowel treatment, infection, and blood transfusion. Factors that protect against postoperative ileus include early enteral nutrition, minimally invasive surgery, and multimodal pain treatment. Interventions that can shorten or prevent such ileus include minimally invasive surgery, early enteral nutrition as well as use of chewing gum, laxatives, and alvimopan. Most of these interventions have been integrated into current guidelines for enhanced recovery of gastrointestinal function after surgery. Future high-quality research is needed in order to clarify our understanding of efficacy and safety.

## 1. Introduction

Abdominal tumors include gastrointestinal tumors, hepatobiliary pancreatic tumor, tumors of urinary system, and gynecological tumors.^[1–5]^ Surgical excision is one of the main treatments for many abdominal tumors. Even after operations that do not involve the gastrointestinal tract, patients often recover gastrointestinal function slowly. Gastrointestinal dysfunction after surgery can include postoperative ileus (POI), which can manifest as nausea, vomiting, abdominal distension, difficulty in defecation, and food intolerance. POI involves nonmechanical suppression of gastrointestinal motility, leading to accumulation of gastrointestinal secretions and in turn to bloating and vomiting.

In long-term cases, patients with POI cannot eat and require parenteral nutrition support. POI reduces patient comfort, prolongs hospitalization after surgery, as well as increases the incidence of perioperative complications and rehospitalization, all of which can substantially increase medical costs.^[6]^

These considerations highlight the need to understand clinicodemographic factors that may increase risk of POI and to identify interventions that can mitigate or prevent it. Therefore the present review comprehensively reviewed the relevant research literature to address these questions.

## 2. Literature search

PubMed database (https://pubmed.ncbi.nlm.nih.gov) was searched using the keywords “intestinal obstruction” and “postoperative ileus” to identify relevant research studies in English published through May 2024. Randomized controlled trials (RCTs), large cohort studies, meta-analyses, and systematic reviews were considered. RCTs and meta-analyses that were high-quality according to standard criteria of Cochrane risk bias assessment tool for RCTs and AMSTAR 2 for meta-analysis^[7]^ were gave priority. Reference lists in meta-analyses and systematic reviews were manually searched. Abstracts, case reports or papers published in languages other than English were not considered.

We also searched for guidelines for enhanced recovery after surgery (ERAS) in the PubMed database on May 2024 using the search string “(Guidelines[Title/Abstract]) AND (Enhanced Recovery After Surgery[Title/Abstract]).” At the same time, I searched for ERAS guidelines on the website https://erassociety.org/guidelines/. Only the latest version of published guidelines were considered in this review.

## 3. Definition and diagnosis of POI

Normally, the small intestine recovers function within 0 to 24 hours after surgery; the stomach, within 24 to 48 hours; and the colon, within 48 to 72 hours.^[8,9]^ There is no universal definition of POI. An international expert consensus has concluded that POI is a temporary, nonmechanical symptom that occurs after surgical intervention and prevents the patient from eating normally. Disappearance of peristalsis can lead to the accumulation of gastrointestinal secretions in the intestinal cavity, poor exhaust, inability to pass stool; these conditions manifest as abdominal pain, symmetrical abdominal distension, anorexia, nausea or vomiting. The 3 most frequent clinical signs of POI are abdominal distension, tenderness, and absence of normal bowel sounds,^[10]^ which are similar to signs of early postoperative small bowel obstruction, making POI sometimes difficult to diagnose.

Primary POI has been defined as temporary cessation of gastrointestinal coordination after surgical intervention that prevents effective transport of gastrointestinal contents, or that renders the patient intolerant to oral ingestion. Secondary POI has been defined as POI in the presence of other definite complications, such as sepsis and anastomotic leakage.^[9]^ Recurrent POI is defined as recurrence of POI after brief remission.

There is no consensus distinction between POI and prolonged postoperative ileus (PPOI). A systematic review of 52 studies and global online survey^[11]^ defined POI as symptoms lasting 1 to 7 days (median: 4 days) from surgery until exhaust or defecation and tolerance of an oral diet. One study has suggested that POI should be defined as symptoms lasting >3 days if assessed using laparoscopy or 5 days if assessed using laparotomy.^[8]^ PPOI, in contrast, is defined as the time from surgery until at least 2 of the following occur: nausea or vomiting, inability to tolerate an oral diet during the past 24 hours, no exhaust during the past 24 hours, abdominal distention, and radiographic confirmation of intestinal obstruction.^[11]^

Both abdominal X-ray imaging and computed tomography can be used to diagnose POI, although computed tomography appears to be more accurate for distinguishing mechanical ileus from POI and it can reveal intra-abdominal abscess or anastomotic leakage that allow differentiation of primary and secondary POI.^[12]^ Imaging with water-soluble contrast agent can help diagnose POI when clinical signs and abdominal X-ray imaging are inconclusive.^[10]^

## 4. Incidence of POI and impact on medical costs

While POI is known to be a frequent complication after surgeries to treat many abdominal or pelvic diseases, reported incidence varies widely in the literature, reflecting differences in definitions and patient populations. One study found that POI occurred in 17% of patients after colectomy, prolonging hospitalization by 29% and increasing medical costs by 15%.^[13]^ Another study reported an incidence of 24% among patients who underwent colectomy, with an associated increase in medical costs.^[6]^ A study involving 160 hospitals in the US found an incidence of 19% of patients after abdominal surgery, lengthening hospitalization and medical costs.^[14]^ A much higher incidence of 34.9% was reported among patients who underwent colorectal surgery, and POI significantly increased the incidence of complications and medical costs.^[15]^ Other studies have reported incidences of POI ranging from 10 to 30%.^[16,17]^

## 5. Risk factors of POI

Numerous clinical studies have reported independent risk factors of POI^[18–32]^: smoking, male, advanced age, cardiac complications, previous history of abdominal surgery, open surgery, fistula, repeated surgery, prolonged operation, long-term opioid analgesia, preoperative hypoalbumin, obesity, conversion to open surgery, intraperitoneal surgical site infection, peritonitis, preoperative electrolyte disturbance, repeated intestinal treatment, and excessive blood loss (Table 1). A meta-analysis of 54 studies involving 18,983 patients who underwent colorectal surgery found that surgical duration and type of surgery (open or laparoscopic) affected risk of POI,^[17]^ and other work identified another risk factor to be whether the surgery involved the right or left colon.^[35,36]^

**Table 1:** Risk factors of postoperative ileus and potential explanations for the increased risk.

Risk factor	Potential explanation
Smoking	Reduces arterial blood flow and tissue oxygenation in the digestive tract.^[21,26]^
Male sex	Stronger inflammatory response during surgery and higher pain threshold, leading to higher catecholamine release.^[8,30,33]^
Old age, cardiac complications, obesity	Reduce surgical tolerance and delay recovery.^[8,29,33]^
Long-term opioid analgesia	M-opioid receptor stimulation inhibits peristalsis.^[32]^
Open surgery, previous abdominal surgery, repeated surgery, long operation time, conversion to open surgery	Increase release of inflammatory mediators because of abdominal injury^[34]^ and use of stupefacient drugs.^[31]^
Colostomy, repeated intestinal treatment	Lead to intestinal edema and aggravate abdominal injury.^[8,33]^
Preoperative hypoalbumin, electrolyte disturbance and excessive blood loss	Excessive crystal input increases intestinal tissue edema.^[8,28,33]^
Intraperitoneal surgical site infection, peritonitis	Excessive release of inflammatory mediators.^[33]^

## 6. Etiology of POI

The etiology of POI is diverse, complex, and poorly understood. It is known to involve the release of inflammatory mediators at the injured site, which inhibit neural reflexes and drug mechanisms,^[9,32]^ and it can be influenced by fluids, electrolytes, and drugs.^[33,37–39]^ One of the most common causes appears to be treatment-related intestinal inflammation.

Surgical procedures can induce adrenergic motor neuron activity, leading to acute intestinal paralysis.^[40]^ Then, at 3 to 4 hours after the surgery, local inflammatory processes induced by the operation impair the contractile activity of the intestine and also activate inhibitory neural pathways and may trigger inflammation in previously unaffected areas, extensively damaging gastrointestinal motility.^[40,41]^ Macrophages and mast cells residing in the outer muscle layer release nitric oxide and prostaglandin, which inhibit the contractile force of smooth muscle, blocking peristalsis. Enteric management can exacerbate gastrointestinal inflammation, prolonging POI;^[34]^ conversely, laparoscopic surgery can reduce the levels of some inflammatory factors^[42]^ and thereby shorten POI.^[43]^

## 7. Prevention and treatment of POI

Up to 98% of patients with POI achieve spontaneous remission using best supportive care.^[18]^ To reduce risk of POI and shorten it when it occurs, thoracic epidural analgesia can be used to block sympathetic reflexes and reduce the need for postoperative opiates, while laparoscopic surgery can be used to reduce trauma. In order to reduce the risk of pharyngitis and respiratory infection,^[44]^ nasogastric tubes should be avoided unless the patient experiences prolonged emergency surgery, severe peritoneal contamination or large blood loss.^[45,46]^ Treatment of PPOI includes using indented nasogastric tubes to relieve abdominal distension, correcting fluid imbalance with water and electrolytes, and maintaining fluid flow. In the event of secondary POI, the likely causes should be treated, and if appropriate, parenteral nutrition should be given.

Studies have reported a number of specific measures that appear capable of minimizing or even preventing POI, which are described in the subsections below (Table 2).

**Table 2:** Comparison of treatments for preventing or mitigating postoperative ileus in patients after abdominal or pelvic surgery.

Treatment	Studies and patients	Potential explanations of efficacy	Efficacy and safety		
			POI or POI-related outcomes	Postoperative hospitalization	Postoperative morbidity and adverse events
Epidural analgesia	One meta-analysis included 16 RCTs with 806 patients who underwent colorectal surgery;^[47]^ another included 94 RCTs with 5846 patients who underwent abdominal surgery.^[48]^	Reduces inflammation, sympathetic nerve stimulation, and use of opioids.	Shortened duration of POI.^[47,48]^	Shortened stay among those who underwent open surgery.^[48]^	Increased incidence of pruritus, urinary retention and arterial hypotension,^[47]^ but not vomiting or anastomotic leakage.^[48]^
Oral carbohydrates before surgery	Four meta-analyses involving patients who underwent elective surgery.^[49–52]^	Relieve inflammation and reduce insulin resistance.	Shortened time until passage of flatus.^[51]^	Shortened stay among patients for whom hospitalization was expected to exceed 1 day.^[49–52]^	Well tolerated.^[49–52]^
Laparoscopic surgery	One RCT involving 427 patients with colectomy;^[53]^ one meta-analysis included 7 studies with 3630 patients who underwent colorectal surgery.^[54]^	Reduces damage to peritoneal and mesangial muscle and abdominal organs, as well as stimulation of abdominal nerve plexus.	Reduced incidence of POI.^[54]^	Shortened stay.^[53]^	Reduced morbidity.^[53]^
Alvimopan	One meta-analysis included 9 RCTs with 4075 patients who underwent open abdominal surgery;^[55]^ one RCT involved 280 patients who underwent radical cystectomy;^[56]^ one small RCT involved 62 patients who underwent cytoreductive surgery and hyperthermic intraperitoneal chemotherapy.^[57]^	Antagonizes the M-opioid receptor.	Reduced incidence of POI.^[55–57]^	Shortened stay.^[55–57]^	Well tolerated^[55–57]^ and decreased POI-related morbidity.^[55,56]^
Chewing gum	One meta-analysis included 10 RCTs with 970 patients who underwent colorectal surgery;^[58]^ one meta-analysis included 10 RCTs with 1463 patients who underwent gynecological surgery;^[59]^ one RCT involved 162 patients who underwent liver resection.^[60]^	Mimics eating.	Reduced incidence of POI.^[58–60]^	One meta-analysis reported shorter stay,^[59]^ but not other studies.^[58,60]^	Well tolerated^[58–60]^ and decreased morbidity.^[59]^
Acupuncture	One meta-analysis included 18 RCTs with 1413 patients who underwent gastrointestinal surgery.^[61]^	Improves local inflammatory responses in the intestine.	Reduced incidence of POI.^[61]^	Shortened stay.^[61]^	Well tolerated but did not influence morbidity.^[61]^
Coffee	One meta-analysis included 13 RCTs with 1246 patients who underwent abdominal surgery.^[62]^	Dilates vascular smooth muscle and reduces inflammatory responses.	Reduced rate of POI.^[62]^	Shortened stay.^[62]^	Well tolerated but did not influence morbidity.^[62]^
Prucalopride	Two RCTs involving 258 patients who underwent gastrointestinal surgery.^[63,64]^	Improves local inflammatory responses in the intestine.	One RCT reported reduced incidence of POI.^[63]^	One RCT reported shorter stay.^[63]^	Well tolerated but did not influence morbidity.^[63,64]^
Simethicone	One RCT involving 118 patients who underwent colorectal surgery.^[65]^	Causes air bubbles in the gut to coalesce, encouraging gas to escape.	No influence.^[65]^	No influence.^[65]^	Well tolerated but did not influence morbidity.^[65]^
Gastrografin	Two small RCTs involving 129 patients who underwent colorectal surgery.^[66,67]^	Easily mobilizes fluids from the extracellular compartment into the intestinal lumen, thus reducing bowel wall edema.	No influence.^[66,67]^	Not reported.	Well tolerated but did not influence morbidity.^[66,67]^
Laxative	One meta-analysis included 5 RCTs with 416 patients who underwent abdominal surgery.^[68]^	Stimulates peristalsis and contraction of the intestinal wall.	Shortened time until passage of stool.^[68]^	No influence.^[68]^	Did not influence morbidity.^[68]^
Early enteral nutrition	One meta-analysis included 17 RCTs with 1437 patients who underwent lower gastrointestinal surgery;^[69]^ another meta-analysis included 10 RCTs with 1237 patients who underwent colorectal surgery.^[70]^	Modulates immune responses early during systemic inflammation.	Reduced incidence of POI.^[69,70]^	Shortened stay.^[69,70]^	Well tolerated but did not influence morbidity.^[69,70]^

POI = postoperative ileus, RCT = randomized controlled trial.

### 7.1. Preoperative oral carbohydrates

Preoperative carbohydrate intake activates the AMPK pathway to inhibit phosphorylation of mTOR/IRS-1, thereby reducing postoperative insulin resistance.^[71]^ Multiple meta-analyses have found that preoperative carbohydrate intake can significantly shorten hospitalization after major abdominal surgery, alleviate postoperative discomfort, and reduce postoperative insulin resistance.^[49–51]^ Whether preoperative carbohydrate intake can reduce risk of POI remains unclear. One study suggested that it can promote recovery of gastrointestinal function by alleviating postoperative inflammatory response,^[72]^ but 4 meta-analyses did not conclude any effect of preoperative carbohydrate intake on POI.^[49–52]^

### 7.2. Epidural analgesia

A meta-analysis comparing the effects of epidural anesthesia with intravenous opioid analgesia on POI after colorectal surgery found that epidural anesthesia accelerated postoperative recovery of intestinal function, while also reducing pain scores.^[47]^ On the other hand, epidural anesthesia significantly increased the incidence of itchy skin, urinary retention, and arterial hypotension. A Cochrane review of 94 RCTs involving 5846 patients found that epidural anesthesia with or without opioids shortened the time until first intestinal venting and bowel movement compared to postoperative epidural local anesthesia.^[48]^ In addition, epidural anesthesia led to lower postoperative pain and, in the case of open surgery, faster postoperative discharge time, without altering the incidence of vomiting or anastomotic leakage.

Epidural anesthesia may inhibit POI by easing inflammation, weakening sympathetic nerve stimulation, and blocking an afferent pathway to reverse the surgery-induced catabolism of cortisol and secretion of glucagon and catecholamine.^[8]^ It also improves insulin sensitivity^[73]^ and reduces perioperative secretion of cytokines.^[74]^

### 7.3. Type of surgery

An RCT comparing laparoscopic and open colorectal surgery found that laparoscopy was associated with shorter times until gastrointestinal passage, first postoperative exhaust and first postoperative exercise.^[75]^ Another RCT found that laparoscopic surgery was associated with significantly shorter postoperative hospitalization and fewer perioperative complications.^[53]^ However, neither of these studies reported incidence of POI. A meta-analysis of 7 RCTs found that laparoscopy was associated with significantly lower incidence of POI than open surgery in patients with colorectal cancer.^[54]^ A retrospective analysis found that open surgery was an independent risk factor for POI after gastric surgery.^[43]^ The potential superiority of laparoscopic surgery may reflect the smaller incision, which reduces injury to the peritoneum and muscle, as well as reduces pulling and damage to abdominal organs and mesentery, which in turn avoids stimulation of the abdominal nerve plexus.

### 7.4. Alvimopan

Endogenous opioids released directly from the gut after surgical trauma and exogenous opioids used for analgesia can negatively affect intestinal motion by activating M-opioid receptors.^[76]^ Alvimopan is an antagonist of the M-opioid receptor in the peripheral nervous system, and it does not easily cross the blood–brain barrier. A meta-analysis of 9 RCTs involving 4075 patients who underwent open surgery found that alvimopan was significantly better than placebo at accelerating recovery of gastrointestinal function, shortening postoperative hospitalization, and reducing the incidence of perioperative POI-related complications.^[55]^ A placebo-controlled RCT also found that alvimopan improved gastrointestinal function, shortened postoperative hospitalization, and reduced the risk of complications after radical bladder cancer resection.^[56]^ A third RCT showed that alvimopan accelerated recovery of gastrointestinal function in patients undergoing cytopathic/intraperitoneal thermoinfusion chemotherapy.^[57]^ Alvimopan is the only drug approved by the US Food and Drug Administration for promoting recovery of gastrointestinal function after surgery, but it has not been approved by the Chinese Food and Drug Administration and the European Medicines Agency. Moreover, prolonged use of alvimopan, such as for 12 months, may increase the incidence of myocardial infarction.^[77]^

### 7.5. Chewing gum

Chewing gum, by mimicking eating, can stimulate recovery of gastrointestinal function after surgery. After colorectal surgery, hepatectomy or gynecological surgery, chewing gum significantly increased the incidence of intestinal peristalsis, intestinal exhaust and defecation, thereby reducing incidence of postoperative POI and perioperative complications as well as shortening postoperative hospitalization.^[58–60]^

### 7.6. Acupuncture

One RCT demonstrated significantly shorter time until first postoperative intestinal peristalsis and flatus after electric acupuncture than after symptomatic supportive treatment.^[78]^ Another RCT reported significantly shorter times until first postoperative exhaust, defecation, start of a semifluid diet, or start of a solid diet than after electric acupuncture at 4 non-acupoints, as well as significantly lower incidence of POI.^[79]^ In fact, meta-analysis of 18 RCTs involving 1413 patients who underwent gastrointestinal surgery found that postoperative acupuncture significantly promoted first postoperative intestinal motility, intestinal exhaust, and defecation, thereby shortening postoperative hospitalization.^[61]^ An RCT from our research team found that the combination of simo decoction and acupuncture was significantly better than chewing gum at promoting the recovery of intestinal peristalsis, flatus and defecation in hepatocellular carcinoma patients after hepatectomy.^[60]^ How acupuncture promotes the recovery of gastrointestinal function is unclear. It may be related to dampening of local inflammatory responses in the intestine.

### 7.7. Coffee

A recent meta-analysis of 13 RCTs involving 1246 patients who underwent colorectal surgery, cesarean section or gynecological surgeries found that oral coffee after surgery significantly reduced the time until first postoperative defecation, incidence of postoperative POI, and length of postoperative hospitalization.^[62]^ How coffee promotes recovery of gastrointestinal function is unclear, and it may be related to the abundant caffeine and chlorogenic acid in coffee. Caffeine increases production of nitric oxide by endothelial cells, and the signaling molecule dilates vascular smooth muscle cells, promoting postoperative recovery of gastrointestinal function.^[80]^ Chlorogenic acid dampens inflammatory responses by reducing production of tumor necrosis factor-α and interleukin-6 in peripheral blood mononuclear cells.^[81]^ These effects may help explain how caffeine can reduce the occurrence of POI.

### 7.8. Prucalopride

RCTs have shown that prucalopride, a highly selective agonist of serotonin receptor 4, can treat paralytic POI,^[82]^ gastroparesis,^[83]^ and chronic constipation.^[84]^ In a placebo-controlled RCT, prucalopride led to significantly shorter time until first defecation, time until first flatus and postoperative hospitalization than placebo, as well as significantly lower incidence of POI and lower level of C-reactive protein.^[63]^ In contrast, a multicenter, double-blind, placebo-controlled RCT showed that oral prucalopride led to significantly shorter time to first defecation than placebo, without affecting POI incidence or postoperative hospitalization.^[64]^ Studies in mice and patients indicated that preoperative, but not postoperative, prucalopride reduced intestinal inflammation (interleukin-6 and interleukin-8) and shortened postoperative hospitalization by inhibiting cholinergic intestinal neurons.^[85]^

### 7.9. Simethicone

Simethicone can cause air bubbles to accumulate in the intestine and thereby promote gas expulsion, but a multicenter, double-blind, placebo-controlled RCT did not find significant benefit of continuous oral administration of simethicone on time until first peristalsis, time until flatus, or postoperative hospitalization after colorectal surgery.^[65]^

### 7.10. Gastrografin

Gastrografin is a water-soluble contrast agent that can hyperpermeabilize membranes and thereby act like an osmotic laxative, mobilizing fluids from the extracellular compartment into the intestinal lumen, thereby reducing bowel wall edema and improving intestinal motility.^[86]^ Gastrografin is often used to distinguish between functional and mechanical small bowel obstruction. However, meta-analysis of 14 prospective studies suggested that gastrografin does not significantly reduce incidence of POI in patients undergoing colorectal surgery.^[87]^ Similarly, 2 small RCTs involving 129 patients also found it did not perform better than distilled water for shortening time until gastrointestinal functional recovery and hospitalization after colorectal surgery.^[66,67]^

### 7.11. Laxative

Advice on laxative use varies across international ERAS guidelines, with some guidelines citing limited safety data and only lower-level evidence of efficacy. One RCT suggested that oral laxatives worked better than placebo at shortening time until first postoperative defecation among patients who underwent open hysterectomy.^[88]^ An RCT of patients undergoing colorectal surgery found that multimodal laxatives significantly shortened time until first defecation and reduced incidence of POI, without affecting length of postoperative hospitalization.^[89]^ A meta-analysis of 5 RCTs involving 416 patients found that laxatives significantly shortened time until first defecation after abdominal surgery, although there was large statistical heterogeneity among studies and the laxatives did not significantly affect time until postoperative flatus, time until feeding or duration of postoperative hospitalization.^[68]^

On the other hand, an RCT suggested that oral laxatives did not significantly alter time until first postoperative flatus, time until first defecation, or postoperative hospitalization in patients who underwent open colectomy.^[90]^ In another RCT, adding preoperative polyethylene glycol to a postoperative polyethylene glycol regimen did not significantly affect time until postoperative defecation or incidence of postoperative complications.^[91]^

Therefore, although many ERAS guidelines include laxatives (see below), the evidence supporting their use is weak or moderate at best.

### 7.12. Early enteral nutrition

Several RCTs have concluded that early enteral nutrition can significantly reduce the occurrence of POI and shorten postoperative hospitalization, without increasing the risk of anastomotic leakage.^[8,92]^ A meta-analysis of 10 RCTs involving 1237 patients found that early postoperative enteral nutrition significantly promoted first flatus and peristalsis compared to traditional postoperative fasting among patients who underwent colorectal surgery, shortening postoperative hospitalization.^[70]^ Early enteral nutrition has become one of the core tenets of multimodal ERAS (see below).

On the other hand, a multicenter RCT of patients who underwent primary colorectal surgery found that perioperative enteral nutrition did not reduce the incidence of POI or anastomotic leakage, nor did it shorten postoperative hospitalization or postoperative inflammation.^[93]^ A systematic review of 17 RCTs involving 1437 patients who underwent surgery of the lower gastrointestinal tract found that early enteral nutrition reduced postoperative hospitalization, but the statistical heterogeneity among studies was large.^[69]^

## 8. Evidence for anti-POI interventions recommended in guidelines

Our review of the research literature in PubMed shows strong support for the efficacy of epidural analgesia, laparoscopic surgery, alvimopan, chewing gum, acupuncture, drink coffee, and early enteral nutrition (Table 2). In the last 10 years, nearly 30 consensus guidelines about ERAS after abdominal or pelvic surgery have been published. Our review of 17 guidelines^[94–110]^ indicates that all but one^[99]^ recommend early enteral nutrition, 9 recommend chewing gum postoperatively,^[94,97,102,104–106,108–110]^ 6 recommend laxatives,^[94,96,102,105,108,110]^ and 3 recommend alvimopan^[97,104,107]^ (Table 3). In addition, 8 guidelines recommend minimally invasive surgery, such as laparoscopic surgery, instead of open surgery.^[94,97,98,104,105,107,109,110]^ Our review highlights that despite powerful evidence in support of some strategies that can aid recovery of gastrointestinal function, not all ERAS guidelines mention them (Table 3). Moreover, even when guidelines include those strategies, they may not indicate the impact on length of postoperative stay or incidence of adverse events, making decision-making difficult for physicians and patients.

**Table 3:** Recommendations for stimulating bowel movement and for conducting minimally invasive surgery and early enteral nutrition in patients after abdominal or pelvic surgery, based on consensus guidelines following the “enhanced recovery after surgery” approach.

Type of surgery and publication year	Stimulation of bowel movement			Minimally invasive surgery			Early enteral nutrition		
	Recommendations	Evidence level	Strength of recommendation	Recommendations	Evidence level	Strength of recommendation	Recommendations	Evidence level	Strength of recommendation
Liver surgery (2022)^[94]^	Postoperative laxatives, gum chewing, herbal medicine, or decoction might reduce the time to first flatus or stool.	Moderate	Weak	Recommended since it reduces postoperative length of stay and complication rates.	Moderate	Strong	Early oral intake with normal diet is recommended.	High	Strong
Liver transplantation (2022)^[95]^	No evidence-based strategies to prevent postoperative ileus.	Low	Weak	Not mentioned.	Not mentioned.	Not mentioned.	Normal food oral intake and/or enteral nutrition should be started 12–24 hours after liver transplantation, according to patient tolerance.	Very low	Strong
Renal transplantation (2022)^[96]^	Laxatives may assist bowel activity.	Moderate	Strong	Not mentioned.	Not mentioned.	Not mentioned.	Patients can tolerate oral fluids immediately and resume a regular diet after surgery.	Moderate	Strong
Colorectal surgery (2022)^[97]^	Chewing gum, alvimopan	Moderate	Strong	Recommended when the expertise is available and when appropriate.	High	Strong	A regular diet should be offered within 24 hours after surgery.	Moderate	Strong
Abdominal and pelvic surgery (2022)^[98]^	Not mentioned.	Not mentioned.	Not mentioned.	Recommended when the expertise is available and when appropriate.	High	Strong	Oral fluids are recommended as soon as the patient is lucid after surgery, and solids are recommended after 4 hours.	Moderate	Strong
Emergency laparotomy (2022)^[99]^	Not mentioned.	Not mentioned.	Not mentioned.	Not mentioned.	Not mentioned.	Not mentioned.	Not mentioned.	Not mentioned.	Not mentioned.
Bariatric surgery (2021)^[100]^	Not mentioned.	Not mentioned.	Not mentioned.	Not mentioned.	Not mentioned.	Not mentioned.	Clear liquid diet can usually be initiated several hours after surgery.	Moderate	Strong
Neonatal intestinal surgery (2020)^[101]^	Not mentioned.	Not mentioned.	Not mentioned.	Not mentioned.	Not mentioned.	Not mentioned.	Breast milk should be started within 24–48 hours after surgery, when possible.	High	Week
Cytoreductive surgery (2020)^[102]^	Laxatives, prokinetics, coffee, and chewing gum could be indicated, alone or in combination.	Low	Weak	Not mentioned.	Not mentioned.	Not mentioned.	Clear liquid should be provided on the day of surgery, and solid food from postoperative day 1.	Moderate	Strong
Vulvar and vaginal surgery (2020)^[103]^	Not mentioned.	Not mentioned.	Not mentioned.	Not mentioned.	Not mentioned.	Not mentioned.	A regular diet should be provided within the first 24 hours after surgery.	Moderate	Strong
Pancreatoduodenectomy (2019)^[104]^	Chewing gum, alvimopan, mosapride.	Moderate	Weak	Should be performed only at high-volume centers by highly experienced personnel applying standardized protocols to ensure safety.	Moderate	Strong	A normal diet without restrictions should be provided after surgery, according to patient tolerance.	Moderate	Strong
Esophagectomy (2019)^[105]^	Chewing gum, laxatives	Low	Weak	Feasible and safe and seems to be associated with some benefits.	Moderate	Moderate	Early enteral nutrition is beneficial	Moderate	Strong
Cesarean delivery (2019)^[106]^	Chewing gum	Low	Weak	Not mentioned.	Not mentioned.	Not mentioned.	A regular diet should be provided within 2 hours after cesarean delivery.	High	Strong
Gynecologic/oncology (2019)^[107]^	Coffee, alvimopan, liposomal bupivacaine	High	Strong	Recommended for appropriate patients when feasible.	High	Strong	A regular diet should be provided within 24 hours after surgery.	High	Strong
Gastrectomy (2014)^[108]^	Chewing gum, laxatives	Low	Weak	Not mentioned.	Not mentioned.	Not mentioned.	Food and drink should be provided ad libitum from postoperative day 1.	Moderate	Week
Radical cystectomy (2013)^[109]^	Chewing gum, oral magnesium	Moderate	Strong	Recommended when feasible	Low	Strong	Food should be started 4 hours after surgery.	Not mentioned.	Strong
Rectal/pelvic surgery (2012)^[110]^	Chewing gum	Moderate	Strong	Feasible and safe and seems to be associated with some benefits.	Moderate	Strong	Food and drink should be started 4 hours after surgery.	Moderate	Strong
	Laxatives	Low	Weak						

## 9. Discussion and future perspectives

Advances in perioperative care over the past 2 decades, particularly the application of ERAS across various surgical fields, appear to have significantly reduced the incidence and duration of POI after abdominal or pelvic surgery. Understanding which interventions have the greatest effect would help channel health care attention and resources to improve prognosis. While our review suggests adequate evidence of efficacy and safety for some interventions recommended in guidelines, it highlights the need for further research to clarify whether epidural analgesia shortens postoperative hospitalization only for patients undergoing open surgery or also for those undergoing laparoscopic surgery,^[48]^ whether preoperative oral carbohydrates can reduce the risk of POI, and whether prucalopride or laxatives can significantly reduce risk of POI and shorten postoperative hospitalization. The available evidence does not support the use of simethicone or gastrografin to improve recovery of gastrointestinal function. The combination of multiple interventions should be explored as potentially superior to single interventions.

In addition, early mobilization after surgery is also beneficial to promote blood circulation, reduce the incidence of deep vein thrombosis, promote the recovery of gastrointestinal function, and thus promote early postoperative rehabilitation. Multimodal pain management strategies that limit the use of morphine is another key factor in ERAS that contributes to minimizing POI. Multimodal pain management strategies can reduce the patient’s sense of pain and anxiety, so that they can get out of bed as soon as possible. The last but not the least, nasogastric tubes should be avoided except under certain circumstances, such as severe peritoneal contamination, or prolonged emergency surgery.

Cost performance and safety are also important indicators for clinical consideration. Chewing gum is inexpensive and certainly less expensive than laxatives, it is compatible with perioperative use, and it has been associated with few side effects or intolerance issues. Alvimopan is a more expensive alternative to chewing gum, but it has been approved only by the US Food and Drug Administration, not by the Chinese Food and Drug Administration or the European Medicines Agency.

Future research should focus on standardizing the definition of POI and developing consensus risk factors that can be built into nomograms to predict patients at higher risk of POI.^[21,111–113]^

This review has some limitations. First, the quality of some included studies may be low. There is some heterogeneity among different studies. Scond, potential biases may exist in the literature selection process. Lastly, the views and conclusions of this review are based on previously published data.

## 10. Conclusion

POI is a common complication after abdominal tumors surgery, and it can even persist in the longer term as PPOI. Minimally invasive surgery, early enteral nutrition, and multimodal pain treatment regimens are well supported by individual RCTs and meta-analyses, and have been integrated into ERAS guidelines. Other strategies may reduce the occurrence of POI and shorten postoperative hospital stay to some extent, including chewing gum, laxatives, alvimopan, and acupuncture.

## Author contributions

**Conceptualization:** Jian-Hong Zhong.

**Data curation:** Jian-Hong Zhong.

**Formal analysis:** Jian-Hong Zhong.

**Funding acquisition:** Jian-Hong Zhong.

**Investigation:** Jian-Hong Zhong.

**Methodology:** Jian-Hong Zhong.

**Project administration:** Jian-Hong Zhong.

**Resources:** Jian-Hong Zhong.

**Software:** Jian-Hong Zhong.

**Supervision:** Jian-Hong Zhong.

**Validation:** Jian-Hong Zhong.

**Visualization:** Jian-Hong Zhong.

**Writing – original draft:** Gui-Sheng Xie, Liang Ma, Jian-Hong Zhong.

**Writing – review & editing:** Jian-Hong Zhong.
